# Sleep quality problems in Thai medical students

**DOI:** 10.5935/1984-0063.20220023

**Published:** 2022

**Authors:** Papan Thaipisuttikul, Thongthai Theansukont, Ratima Boonmueng, Pattarabhorn Wisajun

**Affiliations:** Faculty of Medicine, Ramathibodi Hospital, Mahidol University, Psychiatry - Bangkok - Bangkok - Thailand.

**Keywords:** Sleep Quality, Daytime Sleepiness, Medical Students

## Abstract

**Objective:**

To explore sleep quality and daytime sleepiness problems in medical students at Ramathibodi Hospital.

**Methods:**

This was a cross-sectional study. Data were collected using questionnaires. The questionnaires included (1) Demographic and personal data, (2) The Pittsburgh Sleep Quality Index (PSQI) for sleep quality problem assessment and (3) The Epworth Sleepiness Scale (ESS) for daytime sleepiness assessment.

**Results:**

Of 691 medical students, 165 (23.9%) completed questionnaires. The prevalence of poor sleep quality and daytime sleepiness were 63.6% and 41.8%, respectively. After adjusted for age and relationship concern, academic concern (prevalence ratio 1.46) and nighttime activity without screen (prevalence ratio 1.43) were associated significantly with sleep quality problem. No factor was associated with daytime sleepiness.

**Discussion:**

Interventions to reduce academic burdens should be promoted to improve sleep problems in medical students. Further studies in larger groups of medical students using both subjective and objective measurements should be done in the future.

## INTRODUCTION

Sleep problems are common. Insomnia, sleep quality problem, and excessive daytime sleepiness are among the most common complaints. Previous studies reported both sleep quality problems and daytime sleepiness among medical students^1^. Medical students are particularly vulnerable due to several risk factors such as academic load, night shifts and psychological stress.

Several indicators can be used to measure sleep quality problems; these include subjective sleep quality, sleep latency, sleep duration, habitual sleep efficiency, sleep disturbances, use of sleep medications and daytime dysfunction. The prevalence of sleep quality problems ranges from 19% to 74%^2,3^. The variation is likely due to different measurement tools, baseline demographic data, and cultural differences^1^. Excessive daytime sleepiness is the probability of falling asleep during daily activities. The prevalence of excessive daytime sleepiness ranges from 30% ^4,5^ using structured scale to 90% using self-report sleeping in class experience^6^. Factors associated with poor sleep can be intrinsic or environmental factors. Intrinsic factors; such as, genetic, medical and mental disorders^1,7^. Several environmental factors were identified to be associated with poor sleep problems, such as academic load^1^, internet and screen time usage^8^, and psychological stress^9^. Some factors can also be interrelated. For example, academic concern can cause sleep problems while sleep problems could also affect academic performance^10^. There are very few studies that focused on sleep problems in medical students in Thailand. One study from a medical school in Northeastern Thailand showed that medical students report poor sleep quality during the clinical year more than the pre-clinical year^11^. Another study from a medical school in the southern part of Thailand found that smartphone and tablet use were associated with sleep problems^12^. Another study explored sleep problems in general Thai students^13^, but did not primarily focus on medical students.

Ramathibodi medical school is one of the leading medical program in the center of Bangkok. The school is well-known for its active learning program and involvement of students in research projects. The objective of this study was to explore sleep quality and daytime sleepiness problems in medical students at Ramathibodi Hospital, including both preclinical and clinical years and factors associated with both problems.

## MATERIAL AND METHODS

This was a cross-sectional study performed in October 2019. Data were collected from medical students year 2 to year 5 at Faculty of Medicine, Ramathbiodi Hospital. Sample size was calculated to be at least 165, using prevalence of sleep quality problem as 70%^3^ and error 7%. We distributed the questionnaires to all medical students after their classes or labs. Medical students were asked to fill out and return the questionnaire to the researcher on the same day. The study protocol was approved by the Institute Review Board (IRB) of Ramathibodi Hospital, Mahidol University.

The questionnaires included (1) Demographic and personal data, (2) The Pittsburgh sleep quality index (PSQI), and (3) The Epworth sleepiness scale (ESS)

Demographic and personal data consisted of age, sex, year, rotation, sleep problems after night shifts, nighttime activities before going to bed, duration of nighttime activities, history of medical/psychiatric illness, history of substance use, any recent specific concern. Nighttime activities before going to bed, duration of nighttime activities and any recent specific concern were assessed by open-ended questions and participants filled out the questions freely by themselves. After received all the information, researchers categorized nighttime activities into 2 groups; screen and non-screen activities. Researchers also grouped specific concern into academic, relationship and family concerns.

The PSQI consists of 19 self-rated questions. The results generate scores 0-3 in each of the seven categories: subjective sleep quality, sleep latency, sleep duration, habitual sleep efficiency, sleep disturbances, use of sleep medication, and daytime dysfunction. The Thai version of PSQI had been validated with good psychometric properties and has been used to study in various populations^14^.

The ESS requires participants to rate his/her probability to fall asleep in eight different situations that most people engage in during their daily lives. The Thai version of ESS has been validated with good psychometric properties and has been used to detect obstructive sleep apnea, narcolepsy and sleep disorders^15^.

We performed statistical analysis using SPSS 18.0 for windows. Descriptive analysis was done to assess prevalence of sleep quality and excessive daytime sleepiness problems. Chi-square or Fisher’s exact test was used to compare categorical data. Paired and independent t-tests or nonparametric tests were used to compare continuous data. We conducted univariate analysis to compare participants with and without sleep quality problems and participants with and without excessive daytime sleepiness. Multivariate analysis was done by poisson regression to confirm factors associated with sleep quality problems. Kendall’s coefficient was done to explore relationship between PSQI score, sleep latency and sleep duration. Spearman rank correlation coefficient was done to explore relationship between academic concern and PSQI subscale.

## RESULTS

Of 691 medical students in year 2-5 at Ramathibodi hospital, one hundred and sixty-five participants (23.9%) signed consent and completed questionnaires (Table 1 Demographic data). The average time to fill out this questionnaire was 10-15 minutes. Participants were predominantly male (58.2%). The mean age was 20.77 (1.68, SD). Approximately 65% of the participants were in the pre-clinical year, while the rest were in the clinical year. Over 67% of the participants spent screen time activities before going to sleep within the range of 0-30 minutes. A small number of participants reported a history of medical illness or depression. The concerns that the participants thought might be associated with their sleep problems were issues regarding academic (18.8%), relationship (6.1%), and family (5.5%). Mean sleep latency was 16.26 minutes (12.55,SD) and mean sleep duration was 6.20 hours (1.00,SD).

**Table 1.** Demographic data.

Characteristics	N (percent) or Mean +/-SD
Male	96 (58.2)
Age	20.77 (1.68)
Year: preclinic (2nd and 3rd)	108 (65.4)
: clinical (4th and 5th)	57 (34.6)
- Surgery	36 (21.8)
- Family medicine	9 (5.5)
- Pediatric	6 (3.6)
- Orthopedic	6 (3.6)
Screen time activities before going to sleep	111 (67.2)
Spending time with activities before going to sleep 0-30 minutes	105 (63.6)
History of medical illness	9 (5.5)
History of depression	3 (1.8)
History of anxiety/bipolar disorder	0 (0)
History of alcohol use (beer)	4 (2.4)
History of smoking	1 (0.6)
Other substance	0 (0)
Academic concern	31 (18.8)
Family concern	9 (5.5)
Relationship concern	10 (6.1)
Mean sleep latency (mins)	16.26 (12.55)
Mean sleep duration (hrs)	6.20 (1.00)

The prevalence of sleep quality problems (PSQI score ≥ 5) was 63.6%. Mean total PSQI score was 5.38 (2.18, SD). Mean scores (range from 0 to 3) (SD) of sleep quality, sleep latency, sleep duration, sleep efficacy, sleep disturbance, use of sleep medication and daytime dysfunction were 0.93 (0.66), 0.66 (0.88), 1.55 (0.68), 0.10 (0.37), 0.90 (0.92), 0.08 (0.37), and 1.16 (0.73) respectively. PSQI score was statistically correlated with sleep latency (r=0.550), but not sleep duration (r=0.131).

Factors associated with sleep quality problems (PSQI score ≥ 5) were reported in table 2. We found younger age (p=0.04), nighttime activities without screen (p=0.00), academic concern (p=0.00) and relationship concern (p=0.01) were significantly associated with sleep quality problems.

**Table 2.** Factors associated with sleep quality problems.

Characteristics	Sleep quality problems ( N=105 )	No sleep quality problems (N=60)	p-value
Age	20.0±3.0	21.0±2.0	0.04*
Male	63 (60%)	33 (55%)	0.62
Pre-clinic	72 (68.57%)	36 (60%)	0.31
Ward rotation:			0.16
-Family medicine	2 (6.1%)	7 (29.2%)	
-Pediatrics	4 (12.1%)	2 (8.3%)	
-Surgery	23 (69.7%)	13 (54.2%)	
-Orthopedics	4 (12.1%)	2 (8.3%)	
Nighttime activities			0.00*
-screen time activities	60 (57.1%)	51 (85.0%)	
-other activities without screen	45 (42.9%)	9 (15.0%)	
Time spend for night- time activities, 0-30 minutes	65 (69.1%)	40 (66.7%)	0.71
History of Medical illness	6 (5.7%)	3 (5.0%)	1.00
History of depression	3 (2.9%)	0 (0%)	0.55
History of alcohol use	4 (3.8%)	0 (0%)	0.30
History of smoking	1 (1.0%)	0 (0%)	1.00
Academic concern	29 (27.6%)	2 (3.3%)	0.00*
Family concern	8 (7.6%)	1 (1.7%)	0.16
Relationship concern	10 (9.5%)	0 (0%)	0.01*
Abnormal epsworth score	45 (42.9%)	24 (40%)	0.75

The prevalence of daytime sleepiness (ESS score ≥ 10) was 41.8%. The average ESS score was 8.83 (4.02, SD). Participants were likely to fall asleep while lying down to rest in the afternoon (32.1%), sitting inactive in a public space (26.1%), sitting and reading (23.0%), and being in a car, while stopping for a few minutes in traffic (14.5%).

Factors associated with daytime sleepiness were reported in table 3. None were statistically significant in association with daytime sleepiness.

**Table 3.** Factors associated with daytime sleepiness.

Characteristics	Daytime sleepiness problems (N=69)	No daytime sleepiness (N=96)	p-value
Age	20.0±2.5	21.0±2.0	0.55
Male	37 (53.6%)	59 (61.5%)	0.34
Pre-clinic	48 (96.6%)	60 (62.5%)	0.41
Ward rotation:			0.24
-Family medicine	4 (19.0%)	5 (13.9%)	
-Pediatrics	0 (0%)	6 (16.7%)	
-Surgery	14 (66.7%)	22 (61.1%)	
-Orthopedics	3 (14.3%)	3 (8.3%)	
Nighttime activities			0.18
-screen time activities	42 (60.9%)	69 (71.9%)	
-other activities without screen	27 (39.1%)	27 (28.1%)	
Time spend for nighttime activities, 0-30 minutes	43 (62.3%)	62 (64.6%)	0.70
History of medical illness	4 (5.8%)	5 (5.2%)	1.00
History of depression	2 (2.9%)	1 (1.0%)	0.57
History of alcohol use	1 (1.4%)	3 (3.1%)	0.64
History of smoking	0 (0%)	1 (1.0%)	1.00
Academic concern	13 (18.8%)	18 (18.7%)	1.00
Family concern	5 (7.2%)	4 (4.2%)	0.49
Relationship concern	6 (8.7%)	4 (4.2%)	0.32

We performed multivariate analysis, by using Poisson Regression with Robust Variance to estimate prevalence ratio of factors associated with Sleep Quality Problems (Table 4). After adjusting for age and relationship concern, academic concern was significantly associated with sleep quality problems, with prevalence ratio of 1.46, 95% confident interval 1.26-1.78 and nighttime activity without screen was also significantly associated with sleep quality problems, with prevalence ratio of 1.43, 95% confidence interval 1.16-1.77.

**Table 4.** Multiple Poisson Regression with Robust Variance to estimate prevalence ratio associated with Sleep Quality Problems.

Factors associated with sleep quality problems	Prevalence ratio (95% conficence interval)	p-value
Age	0.95 (0.87-1.04)	0.3
Relationship Concern	1.14 (0.93-1.40)	0.22
Academic Concern	1.46 (1.21-1.78)	0.00*
Nighttime activity without screen	1.43 (1.16-1.77)	0.001*

Academic concen was statistically significantly correlated with four specific items of PSQI; sleep disturbance (r=0.317), sleep quality (r=0.306), sleep latency (r=0.290), and daytime dysfunction (r=0.239).

## DISCUSSION

The prevalence of sleep quality problems among medical students in our study was 63.6% which was similar to the results of previous studies in Asia and Africa^3,10,16^, but higher than Chinese and Lithuanian studies using the same tool^2,17^. The prevalence of daytime sleepiness was 41.8% which was slightly higher compared with the previous studies using the same measurement^4,5^. The population in this study was predominantly male. Sixty-five percent of participants were pre-clinic students and the rest were clinic students. The average sleep latency time was about 16 minutes and the average sleep duration was 6.2 hours.

Similar to the results of previous studies^1,8^, academic and relationship concerns were the major factors associated with sleep problems. While the difference in preclinical and clinical year was not a significant factor, students with sleep problems tended to be younger. However, after using multivariate to manage confounding factors, academic concern remained a significant factor associated with poor sleep quality. Previous studies^7,12^ reported that internet and screen time usage was associated with poor sleep quality. Surprisingly, our study showed that spending nighttime activities on screen was not associated with poor sleep quality. In our medical school program, early-year medical students can spend time studying on-line lectures at their own pace. We postulate that younger medical students tend to study late at night and consider these activities as their nighttime routine before bedtime. Some students may also using screen on recreational activities before going to bed, for example, talking with friends, watching movies or listening to music. Some of these screen activities may help them relax before sleep. This can also be a limitation of our open-ended question to assess our nighttime activities. Therefore, more detailed on nighttime activity without screen,with more specific questionnaire assessment should be confirmed in larger study, since our study classified only screen vs non-screen activity before going to bed and many previous studies found screen time activity associated with sleep quality problems ^9,12^. This study did not find any factors associated with daytime sleepiness, including sleep quality problems. A slightly higher prevalence compared with previous studies may be related to the different cut-off scale that we used^5^.

Academic concern was the strongest factor associated with poor sleep quality in this study. Sleep disturbance (r=0.317) and subjective sleep quality (r=0.306) were 2 items of PSQI that was moderatedly correlated with academic concern in this study, while other sleep items had weakly positive or no correlation. Academic concern, in our study, affected on sleep as similar to many previous studies^1,8,18^, however, it was less related with use of sleep medication and alcohol or substance use disorder which found to be more significant problems in other college students^18^. Academic concern involve not only academic performance or academic load, but also learning atmosphere and professional value^19^. Our suggestions to help lessen academic concern in medical students are (1) medical students should know their learning objectives (2) medical students should have their own mentors that they can easily share their concerns, promote their value and professionalism^20^.

This study has some noteworthy limitations. First, the assessments of sleep quality and daytime sleepiness in this study were subjective. Our response rate was a little lower than expected (23.9%). Those who did not complete the questionnaires may have more or less sleep quality and excessive daytime sleepiness problems. Second, this was a cross-sectional study. Although we were able to identify some factors associated with poor sleep quality, the causative effects can not be concluded. Finally, our participants were medical students in pre-clinic (year 2-3) and clinic (year 4-5) in Thailand. There is limited generalizability to other medical schools with different cultures and approaches.

## CONCLUSION

This study found that the prevalence of poor sleep quality and daytime sleepiness in Thai medical students were 63.6% and 41.8%, respectively. Younger age, academic concern, relationship concern and nighttime activity without screen before going to bed were associated with poor sleep quality problems in univariate analysis. However, academic concern and nighttime activity without screen remained statistically significant in multivariate analysis. No factor was associated with daytime sleepiness in this study. Easing academic burdens should be among the top priorities to improve sleep problem in medical students. Further comprehensive studies in larger groups of medical students using both subjective and objective measurements should be done in the future.

## LISTS OF ABBREVIATION

PSQI: Pittsburgh Sleep Questionnaire Index
ESS: Epsworth Sleepiness Scale
SD: standard deviation
mins: minutes
hrs: hours
